# Determinants of Ascaridoid Nematode Infection and Anisakis‐Related Zoonotic Exposure Risk in Eastern Mediterranean Gadiformes Fishes

**DOI:** 10.1155/tbed/5392704

**Published:** 2026-02-20

**Authors:** Flavia Occhibove, Alejandro López-Verdejo, Valerio Mazzella, Luigi Maria Cusano, Marialetizia Palomba, Renato Aco-Alburqueque, Simonetta Mattiucci, Laura Núñez-Pons, Mario Santoro

**Affiliations:** ^1^ Department of Integrative Marine Ecology, Stazione Zoologica Anton Dohrn, Naples, 80121, Italy, szn.it; ^2^ Marine Zoology Unit, Cavanilles Institute of Biodiversity and Evolutionary Biology, University of Valencia, Paterna, 46980, Spain, uv.es; ^3^ Department of Integrative Marine Ecology, Stazione Zoologica Anton Dohrn, Ischia Marine Centre, Ischia, Naples, 80077, Italy, szn.it; ^4^ National Biodiversity Future Center, Palermo, 90133, Italy; ^5^ Department of Ecological and Biological Sciences, Tuscia University, Viterbo, 01100, Italy, unitus.it; ^6^ Department of Wellbeing, Health and Environmental Sustainability, Sapienza University of Rome, Rome, 00185, Italy, uniroma1.it

**Keywords:** *Anisakis pegreffii*, European hake, greater forkbeard, *Hysterothylacium aduncum*, *Merluccius merluccius*, *Phycis blennoides*, zoonotic risk assessment

## Abstract

Larvae of ascaridoid nematodes, particularly *Anisakis* spp., are common parasites of commercially important marine fishes and may represent a zoonotic hazard following ingestion of raw or undercooked seafood. We investigated the ascaridoid fauna of the sympatric European hake (*Merluccius merluccius*) and greater forkbeard (*Phycis blennoides*) from the Ionian Sea (Eastern Mediterranean), integrating host biometric and seasonal drivers with molecular identification and quantitative risk assessment (QRA) for the zoonotic *Anisakis pegreffii*. *Anisakis pegreffii* was the dominant species in both hosts, followed by *Hysterothylacium aduncum*; other detected taxa included *A. typica*, *A. ziphidarum*, *Skrjabinisakis physeteris*, and *H. fabri*. In both hosts, the larval abundance exhibited marked seasonal peaks in summer and correlated more strongly with host liver and gonad condition indices, suggesting that seasonality, togheter with host physiological state, rather than size alone, modulates infection levels. Most larvae were found in the visceral non edible parts of the fish, while only a small proportion of these were detected in skeletal muscles (2.6% in hake and 0.6% in forkbeard), primarily in the anterior ventral fillet portion. QRA indicated a low per‐meal probability of anisakiasis from untreated hake (~1 case per 52,609 meals). These findings highlight species‐specific, trophically mediated infection patterns and reinforce that European hake and greater forkbeard represent minor but nonnegligible sources of zoonotic risk. Preventive measures, including immediate evisceration, proper freezing or cooking, and selective fillet trimming, are recommended to minimize human exposure.

## 1. Introduction

Ascaridoid nematodes of the families Anisakidae and Raphidascarididae are among the most prevalent helminth parasites infecting a wide range of seafood worldwide [1–3]. Certain members of the Anisakidae can infect humans, causing gastrointestinal anisakiasis after the consumption of raw or undercooked seafood containing viable third‐stage larvae [1–3]. Although members of the Raphidascarididae are generally regarded as posing lower public health risks, occasional human infections by *Hysterothylacium aduncum* have been documented [4]. Freezing or cooking seafood has been demonstrated to reduce the risk of gastrointestinal infections; however, allergic anisakiasis to the freeze‐ and heat‐stable allergens of Anisakidae may still occur [1, 5, 6].

Beyond food safety concerns, the occurrence of ascaridoid larvae in commercially important fish species represents a major socioeconomic challenge, as consumers often reject visibly parasitized products, leading to loss of market value and reputational damage for the seafood industry [7, 8].

The European hake (*Merluccius merluccius*) is among the most commercially valuable demersal fish species in Europe, ranking as the third most profitable species for Mediterranean fisheries [9], also supporting major fisheries in the Northeast Atlantic and North Sea. According to FAO [9], the Mediterranean fishing area gathers the second largest proportion of the total reported hake landings worldwide. The greater forkbeard (*Phycis blennoides*) is a demersal fish widely distributed throughout the Northeast Atlantic, the Mediterranean, and parts of the North Sea. Although of lower economic value compared to the European hake, the greater forkbeard constitutes an important bycatch and target species in specific Mediterranean areas (e.g., [10]). Its commercial relevance has increased in recent years due to changing exploitation patterns and growing consumer diversification within regional seafood markets, particularly in the Eastern and Central Mediterranean [11–13].

Mediterranean and Northeast Atlantic studies on the European hake have documented high prevalence of ascaridoid nematodes and distinct tissue‐distribution patterns, with larvae of different species showing specific affinities for viscera vs. muscle tissues (e.g., [14]). Parasitological studies across Mediterranean fishing grounds have further revealed spatial and size‐related drivers of *Anisakis pegreffii* distribution, the dominant *Anisakis* species in this region [15–17]. However, an integrated evaluation of host physiology, seasonal patterns, and trophic factors shaping ascaridoid infections and associated zoonotic risk remains lacking.

For the greater forkbeard, the available data are scarcer and limited to western Mediterranean populations (e.g., [10, 18]), with few records from the eastern basin [19]. These studies indicate variability in ascaridoid species composition and in infection levels, but rarely address food safety implications.

Critical gaps still persist concerning (i) comparative ascaridoid assemblages in sympatric Gadiformes from the Eastern Mediterranean; (ii) the influence of host biometric, reproductive, and seasonal drivers on infection patterns; (iii) quantification of larval burdens in edible tissues; and (iv) the translation of parasitological data into quantitative zoonotic risk estimates. To address these gaps, the present study characterizes the ascaridoid fauna of the European hake and the greater forkbeard from an Eastern Mediterranean area applying standardized morphological and molecular approaches. Infection drivers are identified through statistical modeling integrating biometric and ecological covariates, and performing a precautionary quantitative risk assessment (QRA) for the zoonotic *A. pegreffii*, thereby linking parasitological evidence to food safety considerations for fisheries sector.

## 2. Materials and Methods

### 2.1. Biometric and Seasonal Data

A total of 150 European hake and 138 greater forkbeard individuals were obtained between January and December 2023 from off Mirto‐Crosia, Ionian Sea, off the coast of Calabria Region in southern Italy. Sampling was conducted using commercial trawling operations at approximately 400–800 m depth, once every 3 months, for a total of four times to cover the 4 yearly seasons: winter (from January to March); spring (from April to June); summer (from July to September); and autumn (from October to December) [20].

Fish were refrigerated at 4°C and examined for parasites within 12/18 h. During dissection procedures, measures were taken for total length (TL, to nearest 0.1 cm), total weight (TW, to the nearest 0.1 g), eviscerated weight (EW), gonad weight (GW), and liver weight (LW); and sex was determined by gonadal examination at the dissection. Body condition index (BCI) was estimated to compare body conditions among individuals, according to the following equation: BCI = 100 × TW/TL^3^ [21]. Additionally, gonadosomatic index (GSI = GW/TW × 100) and hepatosomatic index (HSI = LW/TW × 100) were estimated.

Seasonal variation in host biometric variables was evaluated using a multivariate analysis of variance (MANOVA), with season as the fixed factor and the variables reported in Table 1 (i.e., TL, TW, EW, GW, LW, BCI, GSI, and HSI) as dependent variables. The MANOVA was fitted using the manova function in R [22], and significance was assessed with Wilks’ lambda statistics. Univariate one‐way ANOVAs were conducted for each separate variable using summary.aov after significant multivariate effect was confirmed, with season as the explanatory factor. Post hoc comparisons among seasons were performed with Tukey’s honest significant difference test using the emmeans function in the emmeans package [23], and *p*‐values from univariate ANOVAs were adjusted for multiple testing using the false discovery rate (FDR) correction.

**Table 1:** Biometrical data of European hake and greater forkbeard examined for ascaridoids larvae from the Ionian Sea of southern Italy.

	Winter	Spring	Summer	Autumn	Total
European hake					
*N*	31	31	40	48	150
Sex	f: 28; m: 3	f: 28; m: 3	f: 7; m: 33	f: 13; m: 33; unknown: 2	f: 122; m: 26; unknown: 2
TL	38.0 ± 3.7 (32–44)	44.4 ± 11.6 (31–98)	38.3 ± 4.9 (28–48)	26.4 ± 3.1 (22–36)	35.7 ± 9.3 (22–98)
TW	408 ± 113 (222–591)	612 ± 260 (205–1223)	405 ± 158 (147–765)	137 ± 53 (78–339)	363 ± 232 (78–1223)
EW	380 ± 111 (208–561)	564 ± 238 (194–1142)	376 ± 146 (140–696)	126 ± 49 (71–314)	335 ± 215 (71–1142)
GW	1.5 ± 1.0 (0.3–6.0)	1.9 ± 1.1 (0.4–4.0)	1.2 ± 1.1 (0.2–6.0)	0.4 ± 0.4 (0.1–2.0)	1.2 ± 1.1 (0.1–6.0)
LW	9.0 ± 4.0 (2.0–16.0)	16.8 ± 8.8 (3.6–41.0)	11.2 ± 7.1 (3.3–37.0)	3.0 ± 1.6 (1.2–9.0)	9.3 ± 7.6 (1.2–41.0)
BCI	0.0073 ± 0.0005 (0.006–0.009)	0.0071 ± 0.0013 (0.001–0.009)	0.0069 ± 0.0005 (0.006–0.009)	0.0072 ± 0.0006 (0.006–0.009)	0.0071 ± 0.0008 (0.001–0.009)
GSI	0.36 ± 0.18 (0.13–0.96)	0.31 ± 0.11 (0.13–0.61)	0.29 ± 0.29 (0.12–1.95)	0.26 ± 0.14 (0.07–0.62)	0.30 ± 0.20 (0.07–1.95)
HSI	2.24 ± 0.84 (0.35–4.08)	2.70 ± 0.86 (1.1–5.73)	2.73 ± 0.97 (0.84–5.37)	2.18 ± 0.70 (1.29–5.12)	2.45 ± 0.87 (0.35–5.73)
Greater forkbeard					
*N*	28	30	37	43	138
Sex	f: 0; m: 28	f: 5; m: 23; unknown: 2	f: 6; m: 31	f: 24; m: 19	f: 35; m: 101; unknown: 2
TL	34.9 ± 2.1 (30–40)	29.6 ± 2.8 (25–39)	32.5 ± 2.5 (26–38)	23.6 ± 2.4 (18–29)	29.6 ± 5.1 (18–40)
TW	356 ± 88 (195–632)	192 ± 65 (108–431)	271 ± 80 (166–517)	96 ± 39 (35–207)	217 ± 118 (35–632)
EW	303 ± 79 (175–508)	165 ± 55 (96–375)	243 ± 63 (152–420)	87 ± 35 (31–192)	189 ± 101 (31–508)
GW	0.7 ± 0.3 (0.2–1.5)	0.3 ± 0.2 (0.1–1.3)	0.4 ± 0.2 (0.1–0.9)	0.1 ± 0.1 (0.1–0.3)	0.3 ± 0.3 (0.1–1.5)
LW	21.7 ± 14.3 (5.1–70.0)	8.9 ± 6.6 (1.1–26.2)	13.4 ± 13.5 (1.3–60.1)	2.7 ± 2.2 (0.4–9.0)	10.8 ± 12.1 (0.4–70.0)
BCI	0.0082 ± 0.0011 (0.004–0.010)	0.0072 ± 0.0004 (0.006–0.008)	0.0078 ± 0.0019 (0.005–0.018)	0.0070 ± 0.0010 (0.006–0.010)	0.0075 ± 0.0013 (0.004–0.018)
GSI	0.20 ± 0.05 (0.07–0.30)	0.18 ± 0.06 (0.07–0.29)	0.13 ± 0.03 (0.05–0.19)	0.07 ± 0.04 (0.01–0.16)	0.13 ± 0.07 (0.01–0.30)
HSI	5.68 ± 2.28 (2.55–11.08)	4.25 ± 2.14 (0.91–9.04)	4.23 ± 2.96 (0.65–11.62)	2.68 ± 1.64 (0.78–7.38)	4.05 ± 2.51 (0.65–11.62)

*Note*: Measurements are expressed as mean ± standard deviation following by range in parenthesis. *N* indicates the number of individuals examined for each season.

Abbreviations: BCI, body condition index; EW, eviscerated weight (in g); f, female; GSI, gonadosomatic index; GW, gonad weight (in g); HIS, hepatosomatic index; LW, liver weight (in g); m, male; TL, total length (in cm); TW, total weight (in g).

### 2.2. Collection of Ascaridoid Nematodes

In the present study, we focused on the collection and identification of larvae of ascaridoid nematodes due to their zoonotic potential. At dissection, each fish was cut along the ventral midline, the viscera were removed, and the body cavity was studied for ascaridoid nematodes. Then, each organ (eyes, gills, heart, gonads, liver, spleen, stomach, and intestine) was individually opened in a Petri dish, filled with water, and examined under a dissecting microscope. When present, ascaridoid nematodes were collected, counted, and the information was recorded for each organ. Subsequently, parasites were preserved in 70% ethanol or frozen (−20°C) for morphological and molecular analyses, respectively [24].

Skeletal muscles were examined individually for larvae of ascaridoid nematodes applying the standard UV‐press method [25]. Before dissection, to localize the distribution of larvae, each fillet was schematically divided into four portions indicated as anterior‐dorsal (AD), posterior‐dorsal (PD), anterior‐ventral (AV), and posterior‐ventral (PV). The right and left fillets of each fish were placed in individual plastic bags, flattened to a 1–2 mm thick layer using a hydraulic press at 5 bar, and frozen for ≥24 h. Fillets were then inspected under a 366 nm UV‐light source for typically fluorescent parasite larvae, which were located, collected, counted, and preserved in ethanol 70% or frozen (−20°C) for subsequent analyses.

Before storage, the anterior extremity of each ascaridoid larva was cut and clarified in Amman’s lactophenol for a generic morphological identification using a compound microscope according to available identification keys [26], while a portion of the middle body tract was used for molecular analysis. Moreover, morphological identification of anisakid third‐stage larvae allowed only broad assignment to morphotypes (e.g., type I vs. type II sensu Berland [26]); therefore, reliable species‐level identification required the application of a multilocus molecular approach, following widely accepted standard procedures [2].

### 2.3. Molecular Identification of Ascaridoid Nematodes

Genomic DNA of all sampled ascaridoid nematodes was extracted using Quick‐gDNA Miniprep Kit (Zymo Research, USA) following the standard manufacturer‐recommended protocol, except for the addition of CTAB buffer (Promega, USA) to the Proteinase K (Thermo Scientific, USA) in the digestion step (incubation for 2 h at 56°C).

First, the mitochondrial cytochrome *c* oxidase subunit II gene (mtDNA *cox*2) was amplified using the primers 211F (5‐TTTTCTAGTTATATAGATTGRTTTYAT‐3) and 210R (5‐CACCAACTCTTAAAATTATC‐3) [27]. Polymerase chain reactions (PCRs) were carried out in a 25 μL volume containing 0.5 μL of each primer 10 μm, 3 μL of MgCl_2_ 25 mM (Promega), 5 μL of 5× buffer (Promega), 0.5 μL of DMSO 0.3 mM, 0.5 μL of dNTPs 10 mM (Promega), 0.3 μL of Go‐Taq Polymerase (5 U/μL) (Promega), and 2 μL of total DNA template, adding ultrapure water to reach the final volume. Thermo‐cycling conditions followed those reported in Palomba et al. [28]. Additionally, the taxonomy of *Anisakis* larvae identified as *A. pegreffii* and *A. simplex* (s.s) by mtDNA *cox*2 were also tested by sequencing the metallopeptidase 10 (nDNA *nas* 10) nDNA locus [29, 30]. This is because polymorphisms and hybridization have been shown to render species identification based solely on the *cox*2 marker unreliable; therefore, this approach was employed to securely discriminate between the two species, which differ in their ecological and zoonotic significance [30]. The *nas* 10 was amplified using the primers nas10F (5‐GATGTTCCTGCAAGTGATTG‐3) and nas10R (5‐CGCTATTAAGAGAGGGATCG‐3). PCR was carried out in a 25 μL volume containing 0.6 μL of each primer 10 μm, 2 μL of MgCl_2_ 25 mM, 5 μL of 5× buffer, 0.6μL of dNTPs 10 mM, 0.2 μL of Go‐Taq Polymerase (5 U/μL), and 2 μL of total DNA template, adding ultrapure water to reach the final volume. Thermocycling conditions followed those reported in Palomba et al. [29].

All successful PCR products were purified using Agencourt AMPure XP (Beckman Coulter, USA), following the standard manufacturer‐recommended protocol. Clean PCR products were submitted to Sanger sequencing from both strands, utilizing the above‐mentioned primers, through an Automated Capillary Electrophoresis Sequencer 3730 DNA Analyzer (Applied Biosystems, USA) using the BigDye Terminator v3.1 Cycle Sequencing Kit (Life Technologies, USA). The obtained contiguous sequences were assembled and edited using Unipro UGENE v51.0 [31]. Sequence identity was checked using BLASTn [32].

### 2.4. Analysis of Ascaridoid Infection Patterns

Prevalence of infection, mean abundance, mean intensity, and total abundance were calculated according to Bush et al. [33]. Species richness was calculated as the number of parasite species present in each fish specimen [33]. All calculations were performed in R [22].

The nonparametric Kruskal–Wallis *H*‐test was used to investigate differences among host sex and sampling season and ascaridoid nematode abundance (tested by genus, and by species). In case of significant results, post hoc analyses were performed using the Dunn’s test implemented in the FSA R package [34]. Potential relationships between host biometric variables (Table 1) and ascaridoid nematode abundance (tested by genus, and by species) were explored using the Pearson’s correlation coefficient. These analyses were performed separately for each host species. All analyses were performed in R [22].

A stepwise regression modeling approach was implemented to investigate which were the drivers of infection, among hosts’ biometric characters (TL, TW, EW, GW, LW, BCI, GSI, and HSI), sex, and sampling season, for the ascaridoid larvae found in the skeletal muscles (pooling both host species). The presence or absence of larvae in the muscle tissue was modeled with a binomial distribution. The models were implemented using the function stepAIC in the R package MASS [35], using bidirectional elimination. Stepwise regression is a step‐by‐step iterative construction of a regression model involving the selection of independent variables to be used in a final model. The function adds or removes potential explanatory variables in succession while testing for statistical significance and fit after each iteration. The metric considered in the best model selection was the Akaike information criterion (AIC) [36].

The prevalence of *A. pegreffii* infections in the two host species across the four sampling seasons was analyzed by fitting a generalized linear mixed model (GLMM) with a binomial error distribution and logit link using the function glmmTMB of the package glmmTMB [37]. Prevalence predictors (fixed effects) were species, sampling season, host biometric variables—summarized using the first axis (PC1) of a principal component analysis of morphological traits (estimated for each species separately), to account for potential effects of size—and all their interactions: *A. pegreffii* prevalence: species × season × PC1 (i.e., biometric variables by species). Model residuals were validated with the DHARMa package [38] to assess fit. Estimated marginal means and pairwise comparisons were obtained with the emmeans function and reported as predicted probabilities (on the response scale, % infected) with 95% confidence intervals. Odds ratios (ORs) were used to quantify between‐species contrasts within each season.

### 2.5. QRA of Zoonotic Risk From European Hake Consumption

A simplified adaptation of the QRA framework developed by Bao et al. [7] was applied to estimate the zoonotic risk associated with consumption of the “hake” fish category [39] in the study area. Briefly, the QRA followed the standard four steps: (1) hazard identification, (2) hazard characterization, (3) exposure assessment, and (4) risk characterization. Step 1 was defined as ingestion of zoonotic anisakid larvae, and step 2 as development of anisakiasis (allergic reactions were not considered, as they were beyond the scope of this study). For exposure assessment (step 3), we used food consumption data at Level 7 of detail (“hakes”) from CREA [39], combined with prevalence and intensity values of *A. pegreffii* obtained in this study, regardless of larval location (i.e., not limited to skeletal muscle), following a precautionary approach. The dose–response relationship was developed using parameters from our data and the community scenario of Bao et al. [7]. To reflect a “worst‐case scenario,” consistent with the precautionary principle typically applied in food safety, we used the highest prevalence and mean intensity observed in the study. We selected the highest value of “hakes” consumption (i.e., acute consumption: 129 g/day/adult) reported in CREA [39], and assumed 100% parasite viability. The mean weight of European hake from our overall sample was used to estimate the number of “hakes” consumed per day, while the mean ID50 (dose required to cause disease in 50% of subjects) was adopted from Bao et al. [7]. The resulting outputs included the probability of disease per untreated (i.e., uncooked) “hake” meal and the estimated number of such meals required to cause a single case of anisakiasis.

## 3. Results

### 3.1. Biometric and Seasonal Data

The summary of host biometric measurements is presented in Table 1. Host sizes and HSI in the European hake changed across seasons, whereas BCI and GSI did not yield differences (Figure S1 and Table S1). In particular, individuals seemed to be larger (longer and heavier) in spring, progressively declining in size over the winter (Figure S1A–C). This pattern was mirrored by the LW, whilst GW was greater in winter and spring, significantly decreasing in summer and autumn (Figure S1D,E). HSI was significantly lower in autumn (Figure S1H).

The greater forkbeard exhibited a different pattern of seasonal variation in size, BCI, GSI, and HSI (Figure S2 and Table S1). In this species, differences among seasons were more pronounced, with length and weight reaching their highest values in winter (Figure S2A–E). BCI also peaked in winter, but was significantly lower in autumn. Both GSI and HSI followed a similar trend, showing a progressive decline from winter toward autumn.

### 3.2. Morphological and Molecular Identification of Ascaridoid Nematodes

Morphological taxonomy approaches allowed the identification of a total of 845 ascaridoid larvae, including 789 anisakid type I larvae, three anisakid type II larvae, and 53 *Hysterothylacium* larvae in the European hake; and a total of 286 ascaridoid larvae, comprising 185 anisakid type I larvae, seven type II larvae, and 94 *Hysterothylacium* larvae in the greater forkbeard. No adult ascaridoids were found in any of the hosts.

Ascaridoid larvae were molecularly identified at the species level according to the 100% similarity match of the obtained mtDNA *cox*2 sequences with those available in the GenBank database.

In the European hake, sequences of mtDNA *cox*2 allowed the identification of 773 specimens of *Anisakis pegreffii*, six of *A. simplex* (s.s.), five of *A. typica* and *A. ziphidarum*, three of *Skrjabinisakis physeteris*, 48 of *Hysterothylacium aduncum*, and five of *H. fabri*. Sequence analysis of the *nas* 10 nDNA locus confirmed 773 *A. pegreffii* and reassigned the specimens previously identified as *A. simplex* (s.s.) to *A. pegreffii*, resulting in a total of 779 *A. pegreffii* specimens.

According to mtDNA *cox*2 sequences, out of the 286 ascaridoid larvae found in the greater forkbeard, 172 were *A. pegreffii*, six were *A. simplex* (s.s.), five were *A. typica*, two were *A. ziphidarum*, seven were *Skrjabinisakis physeteris*, 90 were *H. aduncum*, and four were *H. fabri*. These showed 99%–100% identity with reference entries available in GenBank. Molecular analysis of the *nas* 10 nDNA gene confirmed *A. pegreffii* identifications, and reassigned the specimens previously identified as *A. simplex* (s.s.) to *A. pegreffii*, resulting in 178 *A. pegreffii* specimens. These sequences showed 100% identity with previously deposited sequences available in GenBank.

Consensus sequences of the mtDNA *cox*2 gene of length approximately 600 bp were deposited in GenBank under the following accession numbers: PX725663 (*A. pegreffii*), PX725664 (*A. typica*), PX725665 (*A. ziphidarum*), PX725666 (*S. physeteris*), PX725667 (*H. aduncum*), and PX725668 (*H. fabri*). Consensus *A. pegreffii* for the *nas* 10 gene was of length 451 bp, and deposited in GenBank under accession numbers: PX841740‐41.

### 3.3. Analysis of Ascaridoid Infection Patterns

Prevalence, abundance, and intensity values for each ascaridoid nematode species found in both host species are reported in Table 2. In the European hake, ascaridoid larvae were detected in several visceral organs and in the body cavity, whereas only 22 larvae (identified as *A. pegreffii*) were recovered from skeletal muscles, with 68% of these found in the AV portion of fillets (Figure 1A and Table 3). *Anisakis pegreffii* was by far the most prevalent and abundant parasite occurring predominantly in the liver and body cavity, while *H. aduncum* was mainly recovered from the intestine and body cavity (Table 3). Although *A. pegreffii* was the dominant taxon, *H. aduncum* also reached relatively high prevalence and abundance values (Table 2).

Figure 1Schematic distribution of ascaridoid larvae in the four muscle sections of the (A) European hake and (B) greater forkbeard. Gray ascaridoid larvae: *H. aduncum*; black larvae: *A. pegreffii*. AD, anterior dorsal; AV, anterior ventral; PD, posterior dorsal; PV, posterior ventral.

**Figure:**
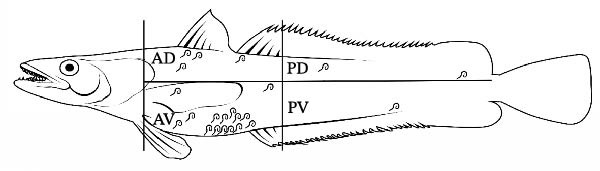
(A)

**Figure:**
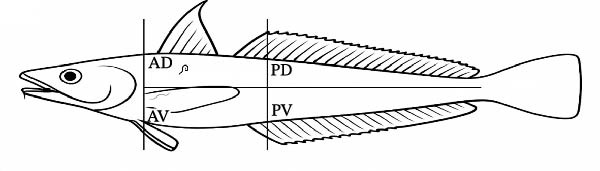
(B)

**Table 2:** Prevalence (P in %), abundance (Ab), and intensity (In) of helminth infections in the European hake and greater forkbeard from the Ionian Sea of southern Italy.

	Winter			Spring			Summer			Autumn			Total		
*N*	31			31			40			48			150		
	P	Ab	In	P	Ab	In	P	Ab	In	P	Ab	In	P	Ab	In
European hake															
* A. pegreffii*	83.9	2.45 ± 2.13 (0–7)	2.92 ± 2.00 (1–7)	90.3	4.87 ± 4.12 (0–14)	5.39 ± 3.99 (1–14)	100.0	8.55 ± 9.71 (1–60)	8.55 ± 9.71 (1–60)	83.3	4.37 ± 6.44 (0–30)	5.25 ± 6.73 (1–30)	89.3	5.19 ± 6.85 (0–60)	5.81 ± 6.99 (1–60)
* A. typica*	3.2	0.03 ± 0.18 (0–1)	1.00 (1–1)	3.2	0.03 ± 0.18 (0–1)	1.00 (1–1)	5.0	0.05 ± 0.22 (0–1)	1.00 ± 0.00 (1–1)	2.1	0.02 ± 0.14 (0–1)	1.00 (1–1)	3.3	0.03 ± 0.18 (0–1)	1.00 ± 0.00 (1–1)
* A. ziphidarum*	6.4	0.06 ± 0.25 (0–1)	1.00 ± 0.00 (1–1)	6.4	0.06 ± 0.25 (0–1)	1.00 ± 0.00 (1–1)	2.5	0.03 ± 0.16 (0–1)	1.00 (1–1)	—	—	—	3.3	0.03 ± 0.18 (0–1)	1.00 ± 0.00 (1–1)
* S. physeteris*	—	—	—	9.7	0.10 ± 0.30 (0–1)	1.00 ± 0.00 (1–1)	—	—	—	—	—	—	2.0	0.02 ± 0.14 (0–1)	1.00 ± 0.00 (1–1)
* H. aduncum*	6.4	0.06 ± 0.25 (0–1)	1.00 ± 0.00 (1–1)	19.3	0.26 ± 0.63 (0–3)	1.33 ± 0.82 (1–3)	20.0	0.35 ± 0.77 (0–3)	1.75 ± 0.71 (1–3)	27.1	0.50 ± 1.50 (0–10)	1.85 ± 2.48 (1–10)	19.3	0.32 ± 0.99 (0–10)	1.66 ± 1.72 (1–10)
* H. fabri*	3.2	0.03 ± 0.18 (0–1)	1.00 (1–1)	9.7	0.10 ± 0.30 (0–1)	1.00 ± 0.00 (1–1)	—	—	—	2.1	0.02 ± 0.14 (0–1)	1.00 (1–1)	3.3	0.03 ± 0.18 (0–1)	1.00 ± 0.00 (1–1)

*N*	28			30			37			43			138		
	P	Ab	In	P	Ab	In	P	Ab	In	P	Ab	In	P	Ab	In
Greater forkbeard															
* A. pegreffii*	21.4	0.39 ± 1.03 (0–5)	1.83 ± 1.60 (1–5)	16.7	0.17 ± 0.38 (0–1)	1.00 ± 0.00 (1–1)	62.2	3.92 ± 6.42 (0–28)	6.30 ± 7.19 (1–28)	34.9	0.39 ± 0.58 (0–2)	1.33 ± 0.35 (1–2)	35.5	1.29 ± 3.71 (0–28)	3.63 ± 5.52 (1–28)
* A. typica*	—	—	—	3.3	0.03 ± 0.18 (0–1)	1 (1–1)	8.1	0.08 ± 0.28 (0–1)	1 ± 0 (1–1)	—	—	—	3.6	0.04 ± 0.19 (0–1)	1.00 ± 0.00 (1–1)
* A. ziphidarum*	—	—	—	3.3	0.03 ± 0.18 (0–1)	1 (1–1)	2.7	0.03 ± 0.16 (0–1)	1 (1–1)	—	—	—	1.4	0.01 ± 0.12 (0–1)	1.00 ± 0.00 (1–1)
* S. physeteris*	25.0	0.25 ± 0.44 (0–1)	1 ± 0 (1–1)	—	—	—	—	—	—	—	—	—	5.1	0.05 ± 0.22 (0–1)	1.00 ± 0.00 (1–1)
* H. aduncum*	—	—	—	30.0	0.63 ± 1.27 (0–5)	2.11 ± 1.54 (1–5)	37.8	1.46 ± 4.32 (0–24)	3.86 ± 6.45 (1–24)	20.9	0.4 ± 0.95 (0–5)	1.89 ± 1.27 (1–5)	23.2	0.65 ± 2.41 (0–24)	2.81 ± 4.40 (1–24)
* H. fabri*	—	—	—	6.7	0.07 ± 0.25 (0–1)	1 ± 0 (1–1)	2.7	0.03 ± 0.16 (0–1)	1 (1–1)	2.3	0.02 ± 0.15 (0–1)	1 (1–1)	2.9	0.03 ± 0.17 (0–1)	1.00 ± 0.00 (1–1)

*Note: N* indicates the number of individuals examined for each season; Ab and In are expressed as mean ± standard deviation following by range in parenthesis.

**Table 3:** Counts of ascaridoid larvae recovered from different localization of the European hake and greater forkbeard from the Ionian Sea of southern Italy.

	Cavity	Gills	Gonad	Intestine	Liver	Sk. muscle	Stomach
European hake							
* A. pegreffii*	233	15	29	51	294	22	135
* A. typica*	1	—	—	—	3	—	1
* A. ziphidarum*	1	—	1	—	1	1	1
* S. physeteris*	1	—	—	—	1	—	1
* H. aduncum*	23	5	—	13	—	—	7
* H. fabri*	2	—	1	—	2	—	—
Total	255	20	31	64	301	29	145
Greater forkbeard							
* A. pegreffii*	21	—	1	25	46	1	84
* A. typica*	1	—	—	1	1	—	2
* A. ziphidarum*	1	—	—	—	—	—	1
* S. physeteris*	1	—	—	2	—	—	4
* H. aduncum*	9	—	—	33	—	1	47
* H. fabri*	—	—	—	—	4	—	—
Total	33	0	1	61	51	2	138

*Note:* Values indicate the number of individual parasites observed per tissue/organ for each taxon.

Abbreviation: Sk. muscle, skeletal muscle.

Seasonal analysis revealed that in the European hake, *A. pegreffii* abundance showed significant differences among seasons (*H* = 26.4; df = 3; *p*  < 0.001), being significantly more abundant in summer (Figure 2A); the same pattern was observed for the overall abundance of *Anisakis* spp. (*H* = 26.1; df = 3; *p*  < 0.001) (Figure 2B). *Skrjabinisakis physeteris* was only recorded in spring (*H* = 11.7; df = 3; *p* = 0.01). The most abundant species, *A. pegreffii*, showed positive correlations with GW, LW, GSI, and HSI. Similarly, *A. ziphidarum* was positively correlated with GW, while *S. physeteris* correlated with TW and EW (Figure 3A). Overall, the abundance of *Anisakis* spp. followed the same pattern observed for *A. pegreffii* (Figure 3A).

Figure 2Box and whisker plots showing variation in the abundance of (A) *A. pegreffii* and (B) total abundance of *Anisakis* spp. in the European hake, and abundance of (C) *A. pegreffii* and (D) *H. aduncum* in the greater forkbeard across the four sampling seasons. Lower and upper box boundaries are 25^th^ and 75^th^ percentiles, respectively; line inside box is the median; lower and upper error lines are 10^th^ and 90^th^ percentiles, respectively; filled circles indicate outliers. Different lowercase letters above boxes denote significant pairwise differences between seasons based on post hoc tests (*p*  < 0.05); groups sharing the same lowercase letter are not significantly different.

**Figure:**
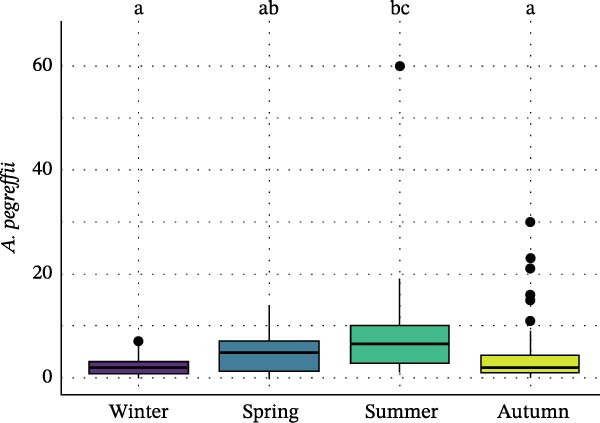
(A)

**Figure:**
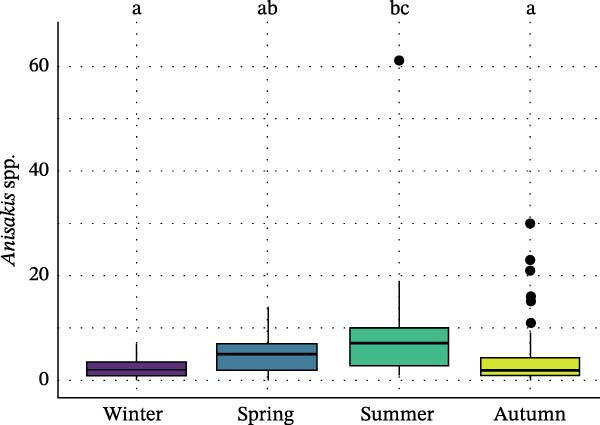
(B)

**Figure:**
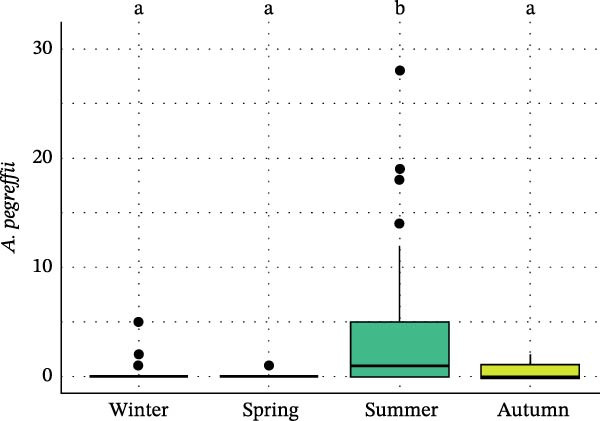
(C)

**Figure:**
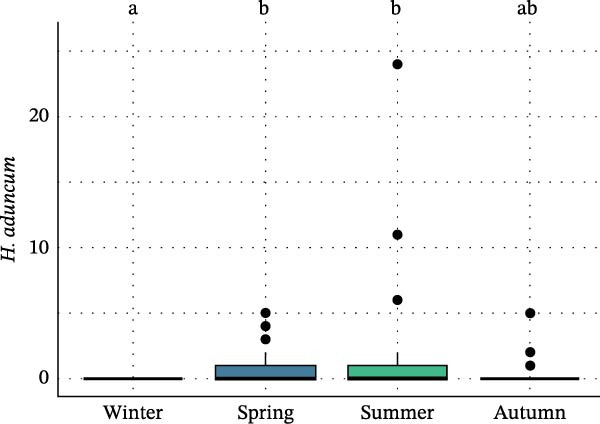
(D)

Figure 3Pearson correlation matrix between parasite abundances, *A. pegreffii* (Ap), *A. typica* (At), *A. ziphidarum* (Az), *S. physeteris* (Sp), *H. aduncum* (Ha), *H. fabri* (Hf), *Anisakis* spp. (Anis), and *Hysterothylacium* spp. (Hyst), and host biometric variables (TL, TW, EW, GW, LW, BCI, GSI, and HSI) in (A) European hake and (B) greater forkbeard. Color indicates strength and direction of correlations (see legend), while blank cells represent nonsignificant correlations (*p* ≥ 0.05). Significant correlations are annotated with their exact coefficients and significance stars: *p*  < 0.05 ( ^∗^), *p*  < 0.01 ( ^∗∗^), and *p*  < 0.001 ( ^∗∗∗^).

**Figure:**
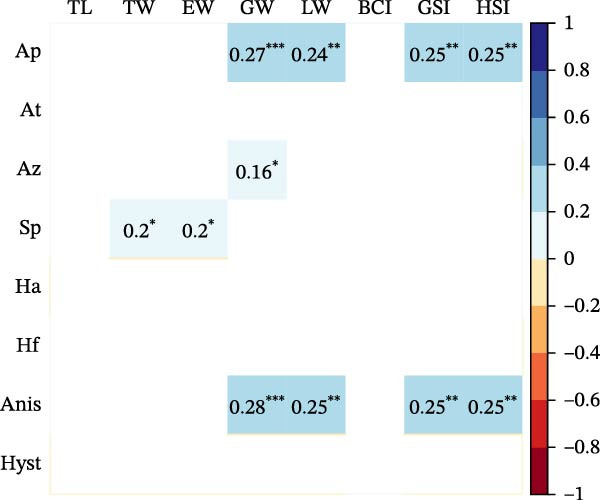
(A)

**Figure:**
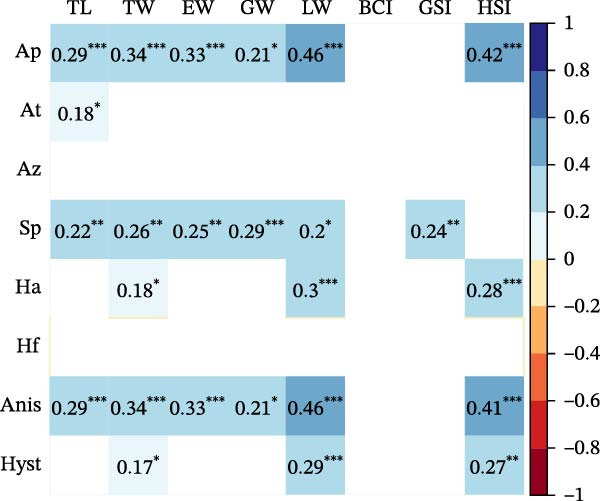
(B)

In the greater forkbeard, only two larvae (identified as *A. pegreffii* and *H. aduncum*, respectively) were recovered in skeletal muscle (Figure 1B and Table 3). In this host, *A. pegreffii* again predominated, with the highest counts reported in the stomach and liver (Table 3), while *H. aduncum* was primarily associated with the gastrointestinal tract, and *H. fabri* was restricted to the liver. Significant seasonal variation in ascaridoid abundance was further recorded, with *A. pegreffii* peaking in summer (*H* = 26.6; df = 3; *p*  < 0.001; Figure 2C). In contrast, *S. physeteris* peaked in winter (*H* = 28.8; df = 3; *p*  < 0.001), while *H. aduncum* reached highest abundances in spring and summer (*H* = 13.3; df = 3; *p* = 0.004; Figure 2D). Total *Anisakis* abundance (*H* = 26.1; df = 3; *p*  < 0.001) and total *Hysterothylacium* abundance (*H* = 13.0; df = 3; *p* = 0.005) also varied significantly across seasons, following the same patterns as their most abundant species, *A. pegreffii* and *H. aduncum*, respectively. The abundance of *A. pegreffii* was correlated with all biometric variables except BCI and GSI, mirroring the pattern for the total abundance of *Anisakis* spp. (Figure 3B), while *A. typica* correlated only with TL (Figure 3B). *Skrjabinisakis physeteris* was positively correlated with all biometric variables except BCI and HSI, while *H. aduncum* was correlated with TW, LW, and HSI (Figure 3B).

*Anisakis pegreffii* prevalence varied markedly between host species and across seasons (Table 4). In winter, predicted prevalence was higher in European hake than in greater forkbeard, but despite the elevated odds of infection, the difference was not statistically significant (OR = 8.0, *p* = 0.0898) (Table 4). In spring, prevalence reached 93.3% in European hake compared to only 12.2% in greater forkbeard; here, the odds of infection were nearly 100‐fold higher in hake, and the difference was highly significant (OR = 99.0, *p* = 0.0005) (Table 4). By summer, nearly all European hake were infected (100%, model‐saturated estimate), making the species contrast inestimable; however, prevalence in greater forkbeard remained below 50%, yielding a conspicuous contrast between the two fish. In autumn, prevalence stayed very high in European hake, with odds of infection approximately 92 times higher than in greater forkbeard (*p* = 0.0052) (Table 4). Substancially, biometric variables and their interactions were not significantly valid predictors of prevalence, as represented in PC1. Overall, European hake consistently exhibited high *A. pegreffii* prevalence across all seasons, with significantly higher infection rates than greater forkbeard in spring, summer, and autumn.

**Table 4:** Predicted prevalence (%) of *A. pegreffii* infections in the two host species, European hake and greater forkbeard, across the four sampling seasons, with 95% confidence intervals (CIs), odds ratios (ORs) for European hake (MM) vs. greater forkbeard (PB), and *p*‐values from pairwise contrasts.

Sampling season	MM prevalence % (95% CI)	PB prevalence % (95% CI)	Odds ratio (MM/PB)	*p*‐Value
Winter	78.5 (55.2–91.5)	31.9 (5.3–79.5)	8.0	0.0898
Spring	93.3 (59.2–99.2)	12.2 (3.9–32.4)	99.0	0.0005
Summer	100 (fixed)	43.9 (24.5–65.5)	—	—
Autumn	98.3 (75.7–99.9)	38.9 (15.8–68.2)	91.9	0.0052

*Note*: Estimates are back‐transformed from the logit scale.

Stepwise negative binomial regression modeling revealed that the presence of *A. pegreffii* larvae in the skeletal muscle of either host species did not seem to be correlated to any of the predictors. Namely, the probability of finding an *A. pegreffii* larva in the skeletal muscle in a particular individual could not be predicted by any of the variables included in the model.

### 3.4. QRA of Zoonotic Risk From European Hake Consumption

For QRA purposes, the estimated dose of *A. pegreffii* ingested in a single meal of untreated hake resulted 1.1, based on: (i) a mean TW of 363 g (equivalent to ~ 0.3 hake per meal); (ii) the highest *A. pegreffii* prevalence observed in this study (100%, irrespective of location, not limited to skeletal muscle); and (iii) the highest mean intensity (8.5) of *A. pegreffii* (Table 2). According to the present QRA, this dose corresponds to a disease probability of 0.000053, equivalent to one case per 52,609 meals containing untreated hake.

## 4. Discussion

The present study revealed the presence of a rich ascaridoid fauna in two sympatric, commercially important Gadiformes species from the Eastern Mediterranean, with the zoonotic *A. pegreffii* dominating the ascaridoid assemblages in both hosts. The ascaridoids identified comprised larvae belonging to three genera (*Anisakis*, *Skrjabinisakis*, and *Hysterothylacium*) and six distinct species. Some specimens were initially identified as *A. simplex* (s.s.) based on the mitochondrial *cox*2 marker; however, the use of an additional nuclear marker allowed their assignment to *A. pegreffii*. This discrepancy, already documented in the literature, is attributable to mitochondrial introgression, likely resulting from past and/or paleo‐introgression events [30, 40].

All the parasite taxa reported complete their life cycles in odontocetes (*Anisakis* and *Skrjabinisakis*) or teleost fish (*Hysterothylacium*). Crustaceans serve as first intermediate hosts, and fish and squid as second intermediate or paratenic hosts, exhibiting some ecological differences depending on the species. For instance, among anisakids, due to host–parasite co‐speciation events and host feeding ecology, *A. pegreffii* and *A. typica* exhibit host preference for oceanic dolphins (Delphinidae), *A. ziphidarum* for beaked whales (Ziphiidae), and *S. physeteris* for sperm whales (Physeteridae) [2, 17, 41]. The dominance of *A. pegreffii* in the two Gadiformes here examined implies that these fish represent important food items for oceanic dolphins. For *H. aduncum*, only adult forms had been described in Gadiformes [42], therefore, the larval detection reported here suggests early infection. Instead, *H. fabri* is considered a complex of at least three sibling species with limited host specificity [43].

While the component ascaridoid community was similar between the two host species, infection levels differed markedly. The European hake and the greater forkbeard are both demersal, active predators; however, they exhibit distinct feeding preferences. The diet of sub‐adult and adult European hake primarily consists of fish, whereas the greater forkbeard mainly feeds on benthic crustaceans such as decapods and mysids [10, 18, 44]. These trophic differences could explain the contrasting levels of ascaridoid nematode infection, which in the present study were strongly influenced by the dominance of *A. pegreffii*. A host feeding predominantly on crustaceans is expected to harbor fewer *Anisakis* larvae (and ascaridoids in general), since crustaceans typically exhibit very low prevalence and usually contain only a small number of early‐stage larvae (often one) [45–47]. Conversely, piscivorous species accumulate larvae by consuming multiple infected prey, acting as bioaccumulators throughout the food chain, and thus, sustaining higher parasite burdens.

In greater forkbeard, the relatively high occurrence of *H. aduncum* is consistent with its benthic feeding ecology and the parasite’s known transmission through crustaceans [10, 18, 44]. Moreover, environmental and trophic variability in the region may also contribute to these host‐specific patterns. According to Fiorentino et al. [11], the trophic ecology of this host species is largely influenced by mesoscale variability. Such environmental gradients, together with ontogenetic and seasonal shifts in diet and distribution [11], may further modulate host exposure to infective larvae and explain the observed seasonal and interspecific variation in ascaridoid abundance.

The observed summer peak in the abundance of *A. pegreffii*, along with other ascaridoid nematodes, aligns with the patterns reported by Mladineo and Poljak [15], who observed significantly higher *A. pegreffii* abundance during summer periods across several Adriatic fish species (*Engraulis encrasicolus*, *Sardina pilchardus*, *Merluccius merluccius*, *Merlangius merlangus*, and *Scomber japonicus*). In the present study, though, the seasonal increase of *A. pegreffii* abundance in the European hake was not associated with body size (TL, TW, and EW), but rather with physiological indicators (GW, LW, GSI, and HSI). This suggests that host physiological state, rather than size alone, modulates infection levels, possibly under the influence of seasonal abiotic and ecological factors. Indeed, variables, such as temperature, photoperiod, and food availability, strongly affect fish metabolism, reproduction, and immune function [48], thereby influencing both exposure and susceptibility to parasites. Warmer months typically coincide with the migration of definitive hosts and increased deposition of *Anisakis* eggs, together with plankton blooms and elevated water temperatures that accelerate larval development [15, 49–52]. These processes enhance infection transmission throughout the food web.

The physiological correspondence observed here aligns with the hypothesis that infection abundance may reflect seasonal changes in host energy allocation. Ferrer‐Maza et al. [53] demonstrated that, in the Mediterranean, European hake populations appear to be in equilibrium with their metazoan parasites, with infection levels closely linked to energy reserves and reproductive status rather than reflecting pathological stress. During regenerating and developing phases, hake prioritize energy accumulation in the liver over immune investment, which may explain the positive correlation between liver condition (HSI) and parasite abundance. As feeding intensity increases in warmer months, fish accumulate higher energy reserves but also face greater exposure to infective larvae, a pattern consistent with the “feeding–infection” trade‐off [53]. Furthermore, European hake in the Mediterranean exhibits a protracted spawning season with peaks in winter and summer [54, 55], switching between capital and income breeding strategies depending on food availability [56, 57]. The summer shift toward income breeding, driven by increased prey abundance [58], may thus coincide with enhanced feeding activity and, consequently, higher parasite transmission rates. Considering that similar seasonal patterns and correlations were observed in the greater forkbeard, we can hypothesize that comparable mechanisms may operate in this host as well, although further evaluation of species‐specific biological cycles, spawning periods, and related physiological factors is required.

In the present study, only a small proportion of ascaridoid larvae were found in the edible tissues (i.e., fillets) of their hosts, representing 2.6% and 0.6% of the total larval counts in European hake and greater forkbeard, respectively. All larvae were identified as *A. pegreffii*, except for a single *H. aduncum* larva detected in the greater forkbeard. These results are in agreement with previous Mediterranean surveys, which generally report low muscle infection levels in *A. pegreffii*‐infected fish [16, 59]. For instance, Cipriani et al. [16] referring to the European hake sampled in the Ionian Sea observed that only 1.9% of *A. pegreffii* larvae were located in the muscle, in contrast with our 2.6%, in spite of a much greater level of infection (mean abundance 40.9 vs. 2.35–8.50 in the present study) and host size (mean TW 1002 g vs. 363 g in our sample). Still, the higher percentage of muscle larvae detected in our samples suggests that larval migration into the musculature does not scale linearly with total parasite burden or host size, but may instead depend on other biotic and abiotic factors. In Cipriani et al. [16], samples were immediately frozen, likely limiting post‐mortem larval migration, whereas in our study, fish were refrigerated for up to 18 h. Thus, differences in muscle infection might be attributed to environmental temperature regimes influencing post‐mortem migration. Indeed, Fuentes et al. [14] demonstrated that the time elapsed between capture and analysis affects larval presence in skeletal muscle, as *Anisakis* larvae can migrate from the viscera post‐mortem, particularly in uneviscerated, refrigerated fish. The larval burden observed here likely reflects a combination of the species’ inherent migratory capacity, processing conditions, and host‐ and environment‐related factors. Host size and immune status for instance, can modulate larval distribution: larger fish may harbor more larvae overall, but muscle infection density does not increase proportionally due to greater muscle mass and potential acquired resistance [52]. Similarly, Cruz et al. [60] reported that the proportion of larvae reaching skeletal muscles decreased with increasing fish size, as the migratory distance from viscera to musculature becomes larger. Moreover, intra‐vitam migration of *Anisakis* larvae is often limited to nearby tissues, as indicated by melanized parasitic capsules in the antero‐ventral flesh [61]. Consistent with these findings, most *A. pegreffii* larvae detected in European hake fillets were located in the AV portion, likely reflecting the shortest anatomical route from the peritoneal cavity to the musculature [16, 59, 61].

Results of the QRA yielded an estimated ingested dose of 1.1 *A. pegreffii* larvae per untreated hake meal and a corresponding disease probability of 0.000053 ( ≈ 1 case per 52,609 meals). This very low point estimate of symptomatic anisakiasis risk from a single untreated hake meal is consistent with the low number of larvae found in edible tissues in our study and with other Mediterranean studies that document most *A. pegreffii* larvae allocated in viscera rather than in muscle (e.g., [14, 16, 59]). Importantly, although population‐level genetic structure and intraspecific variability may have epidemiological relevance, the QRA was intentionally parameterized under conservative, worst‐case assumptions, such that potential genetic heterogeneity would not affect risk estimates or the interpretation of results. However, the low estimated clinical risk should be interpreted cautiously. First, QRA models are sensitive to input assumptions (meal size, prevalence in edible tissues, and dose–response), and regional consumption practices that favor raw, marinated, or undercooked fish increase exposure substantially [14, 52]. Second, anisakiasis is considered underdiagnosed in Europe, literature reviews and surveillance data suggest that reported cases underestimate true incidence [7, 8], so population‐level disease burden may be higher than what clinical notifications indicate. Third, allergic reactions to *Anisakis* allergens can occur after ingestion of dead larvae, and thermostable allergens have been demonstrated; these risks are not captured by infection‐only QRA endpoints, and therefore, require separate consideration [14]. Finally, post‐mortem larval migration from viscera to muscle (during delayed chilling, storage or uneviscerated transport) can increase flesh contamination and thus exposure, a factor that can markedly change QRA outcomes if not controlled.

In conclusion, our low per‐meal disease probability indicates a low individual risk under ideal handling/cooking, but nonnegligible public‐health risk remains at the population level where raw/undercooked consumption, poor post‐catch handling, or high local parasite burdens prevail [14, 52]. Targeted, regionally tailored QRA would, therefore, be valuable to refine exposure estimates using local consumption habits and processing chains. From a public health perspective, the increasing detection of *Anisakis* spp. in European markets highlights the importance of preventive measures. Immediate evisceration after capture, proper freezing of raw or lightly processed fish products (−20°C for ≥24 h or −35°C for ≥15 h), and thorough cooking (≥60°C for 5–10 min) remain the most effective control strategies [14]. Additionally, the anatomical distribution of larvae in the skeletal muscle is relevant, as manual trimming and removal of the AV fillet portion can effectively eliminate most muscle‐dwelling larvae, thereby markedly reducing the risk of human anisakiasis, as previously suggested [16, 59, 62]. Consumer education is essential to reduce underdiagnosis and to promote safe preparation practices, particularly given the persistence of thermostable *Anisakis* allergens. Strengthening monitoring procedures and standardizing detection methods at the processing level would further minimize the risk of anisakiasis and improve seafood safety across the supply chain [52].

## Author Contributions

**Flavia Occhibove:** formal analysis, methodology, investigation, visualization, writing – review and editing, writing – original draft. **Alejandro López-Verdejo, Valerio Mazzella, Marialetizia Palomba, Simonetta Mattiucci**, **and Laura Núñez-Pons:** investigation, methodology, writing – review and editing. **Luigi Maria Cusano and Renato Aco-Alburqueque:** investigation. **Mario Santoro:** conceptualization, data curation, funding acquisition, project administration, methodology, investigation, supervision, validation, writing – review and editing, writing – original draft.

## Funding

This study was financed in the frame of the research “Food‐web transmitted endoparasites and their hosts: an integrative approach to investigate the ‘state’ of biodiversity of the marine ecosystem from off Calabria coast,” carried out under the project “Centro Ricerche ed Infrastrutture Marine Avanzate in Calabria (CRIMAC),” funded by FSC 2014–2020 – Piano Stralcio Ricerca e Innovazione 2015–2017 – Programma Nazionale Infrastrutture di Ricerca (PNIR), linea d’azione 1. Cofinanziamento Infrastrutture di Ricerca (IR). Project was also partially supported under the National Recovery and Resilience Plan (NRRP), Mission 4 Component 2 Investment 1.4, Call for Tender Number 3138 of December 16, 2021, rectified by Decree Number 3175 of December 18, 2021 of Italian Ministry of University and Research funded by the European Union—NextGenerationEU: Award Number: Project code CN_00000033, Concession Decree Number 1034 of June 17, 2022 adopted by the Italian Ministry of University and Research, CUP C63C22000520001, Project title “National Biodiversity Future Center (NBFC).”

## Disclosure

All the authors have read and agreed to the published version of the manuscript.

## Ethics Statement

The fish were purchased dead on landing so no permit was required.

## Conflicts of Interest

The authors declare no conflicts of interest.

## Supporting Information

Additional supporting information can be found online in the Supporting Information section.

## Supporting information


**Supporting Information** Table S1. Results of MANOVA and subsequent univariate ANOVAs testing seasonal variation in biometric variables of European hake and greater forkbeard. The MANOVA, with season as the explanatory factor and biometric traits as the response matrix, indicated a significant overall effect of season for both species. Follow‐up one‐way ANOVAs are reported for each variable: total length (TL), total weight (TW), eviscerated weight (EW), gonad weight (GW), liver weight (LW), body condition index (BCI), gonadosomatic index (GSI), and hepatosomatic index (HSI). The table shows degrees of freedom (df), *F* statistics, and associated *p*‐values; significant seasonal effects (*p* < 0.05) are highlighted in bold. Figure S1. European hake box‐and‐whisker plots showing variation in (A) total length (TL), (B) total weight (TW), (C) eviscerated weight (EW), (D) gonad weight (GW), (E) liver weight (LW), (F) body condition index (BCI), (G) gonadosomatic index (GSI), and (H) hepatosomatic index (HSI) across the four sampling seasons. Lower and upper box boundaries are 25^th^ and 75^th^ percentiles, respectively; line inside box is the median; lower and upper error lines are 10^th^ and 90^th^ percentiles, respectively; filled circles indicate individual observations. Different lowercase letters above boxes denote significant pairwise differences between seasons based on post hoc Tukey tests (*p* < 0.05); groups sharing the same letter are not significantly different. Figure S2. Greater forkbeard–box‐and‐whisker plots showing variation in (A) total length (TL), (B) total weight (TW), (C) eviscerated weight (EW), (D) gonad weight (GW), (E) liver weight (LW), (F) body condition index (BCI), (G) gonadosomatic index (GSI), and (H) hepatosomatic index (HSI) across the four sampling seasons. Lower and upper box boundaries are 25^th^ and 75^th^ percentiles, respectively; line inside box is the median; lower and upper error lines are 10^th^ and 90^th^ percentiles, respectively; filled circles indicate individual observations. Different lowercase letters above boxes denote significant pairwise differences between seasons based on post hoc Tukey tests (*p* < 0.05); groups sharing the same letter are not significantly different.

## Data Availability

Data will be made available upon request.
