# Supplementary material for: Determinants of Ascaridoid Nematode Infection and Anisakis‐Related Zoonotic Exposure Risk in Eastern Mediterranean Gadiformes Fishes
Source: Transbound Emerg Dis. 2026 Feb 20;2026:5392704. doi: 10.1155/tbed/5392704 (PMC12921451; doi:10.1155/tbed/5392704)
Supplement: Supplementary file 1 — Supporting Information Table S1. Results of MANOVA and subsequent univariate ANOVAs testing seasonal variation in biometric variables of European hake and greater forkbeard. The MANOVA, with season as the explanatory factor and biometric traits as the response matrix, indicated a significant overall effect of season for both species. Follow‐up one‐way ANOVAs are reported for each variable: total length (TL), total weight (TW), eviscerated weight (EW), gonad weight (GW), liver weight (LW), body condition index (BCI), gonadosomatic index (GSI), and hepatosomatic index (HSI). The table shows degrees of freedom (df), F statistics, and associated p‐values; significant seasonal effects (p < 0.05) are highlighted in bold. Figure S1. European hake box‐and‐whisker plots showing variation in (A) total length (TL), (B) total weight (TW), (C) eviscerated weight (EW), (D) gonad weight (GW), (E) liver weight (LW), (F) body condition index (BCI), (G) gonadosomatic index (GSI), and (H) hepatosomatic index (HSI) across the four sampling seasons. Lower and upper box boundaries are 25th and 75th percentiles, respectively; line inside box is the median; lower and upper error lines are 10th and 90th percentiles, respectively; filled circles indicate individual observations. Different lowercase letters above boxes denote significant pairwise differences between seasons based on post hoc Tukey tests (p < 0.05); groups sharing the same letter are not significantly different. Figure S2. Greater forkbeard–box‐and‐whisker plots showing variation in (A) total length (TL), (B) total weight (TW), (C) eviscerated weight (EW), (D) gonad weight (GW), (E) liver weight (LW), (F) body condition index (BCI), (G) gonadosomatic index (GSI), and (H) hepatosomatic index (HSI) across the four sampling seasons. Lower and upper box boundaries are 25th and 75th percentiles, respectively; line inside box is the median; lower and upper error lines are 10th and 90th percentiles, respectively; filled circles indic [file TBED-2026-5392704-s001.docx]

**Supplementary Material**

Table S1. Results of MANOVA and subsequent univariate ANOVAs testing seasonal variation in biometric variables of European hake and greater forkbeard. The MANOVA, with season as the explanatory factor and biometric traits as the response matrix, indicated a significant overall effect of season for both species. Follow-up one-way ANOVAs are reported for each variable: total length (TL), total weight (TW), eviscerated weight (EW), gonad weight (GW), liver weight (LW), body condition index (BCI), gonadosomatic index (GSI), and hepatosomatic index (HSI). The table shows degrees of freedom (df), F statistics, and associated p-values; significant seasonal effects (p < 0.05) are highlighted in bold.

| European hake | df | F | p |
| --- | --- | --- | --- |
| MANOVA | 24 | 8.9 | **<0.001** |
| TL | 3 | 56.8 | **<0.001** |
| TW | 3 | 60.9 | **<0.001** |
| EW | 3 | 60.6 | **<0.001** |
| GW | 3 | 19.4 | **<0.001** |
| LW | 3 | 36.3 | **<0.001** |
| BCI | 3 | 1.8 | 0.143 |
| GSI | 3 | 1.6 | 0.198 |
| HSI | 3 | 4.5 | **<0.001** |
| Greater forkbeard | df | F | p |
| MANOVA | 24 | 22.9 | **<0.001** |
| TL | 3 | 143.6 | **<0.001** |
| TW | 3 | 92.0 | **<0.001** |
| EW | 3 | 93.2 | **<0.001** |
| GW | 3 | 67.9 | **<0.001** |
| LW | 3 | 21.3 | **<0.001** |
| BCI | 3 | 6.9 | **<0.001** |
| GSI | 3 | 62.9 | **<0.001** |
| HSI | 3 | 10.0 | **<0.001** |

Supplementary figure legends

Figure S1. European hake – Box and whisker plots showing variation in A) total length (TL), B) total weight (TW), C) eviscerated weight (EW), D) gonad weight (GW), E) liver weight (LW), F) body condition index (BCI), G) gonadosomatic index (GSI), and H) hepatosomatic index (HSI) across the four sampling seasons. Lower and upper box boundaries are 25^th^ and 75^th^ percentiles, respectively; line inside box is the median; lower and upper error lines are 10^th^ and 90^th^ percentiles, respectively; filled circles indicate individual observations. Different lowercase letters above boxes denote significant pairwise differences between seasons based on post-hoc Tukey tests (p < 0.05); groups sharing the same letter are not significantly different.

Figure S2. Greater forkbeard – Box and whisker plots showing variation in A) total length (TL), B) total weight (TW), C) eviscerated weight (EW), D) gonad weight (GW), E) liver weight (LW), F) body condition index (BCI), G) gonadosomatic index (GSI), and H) hepatosomatic index (HSI) across the four sampling seasons. Lower and upper box boundaries are 25^th^ and 75^th^ percentiles, respectively; line inside box is the median; lower and upper error lines are 10^th^ and 90^th^ percentiles, respectively; filled circles indicate individual observations. Different lowercase letters above boxes denote significant pairwise differences between seasons based on post-hoc Tukey tests (p < 0.05); groups sharing the same letter are not significantly different.


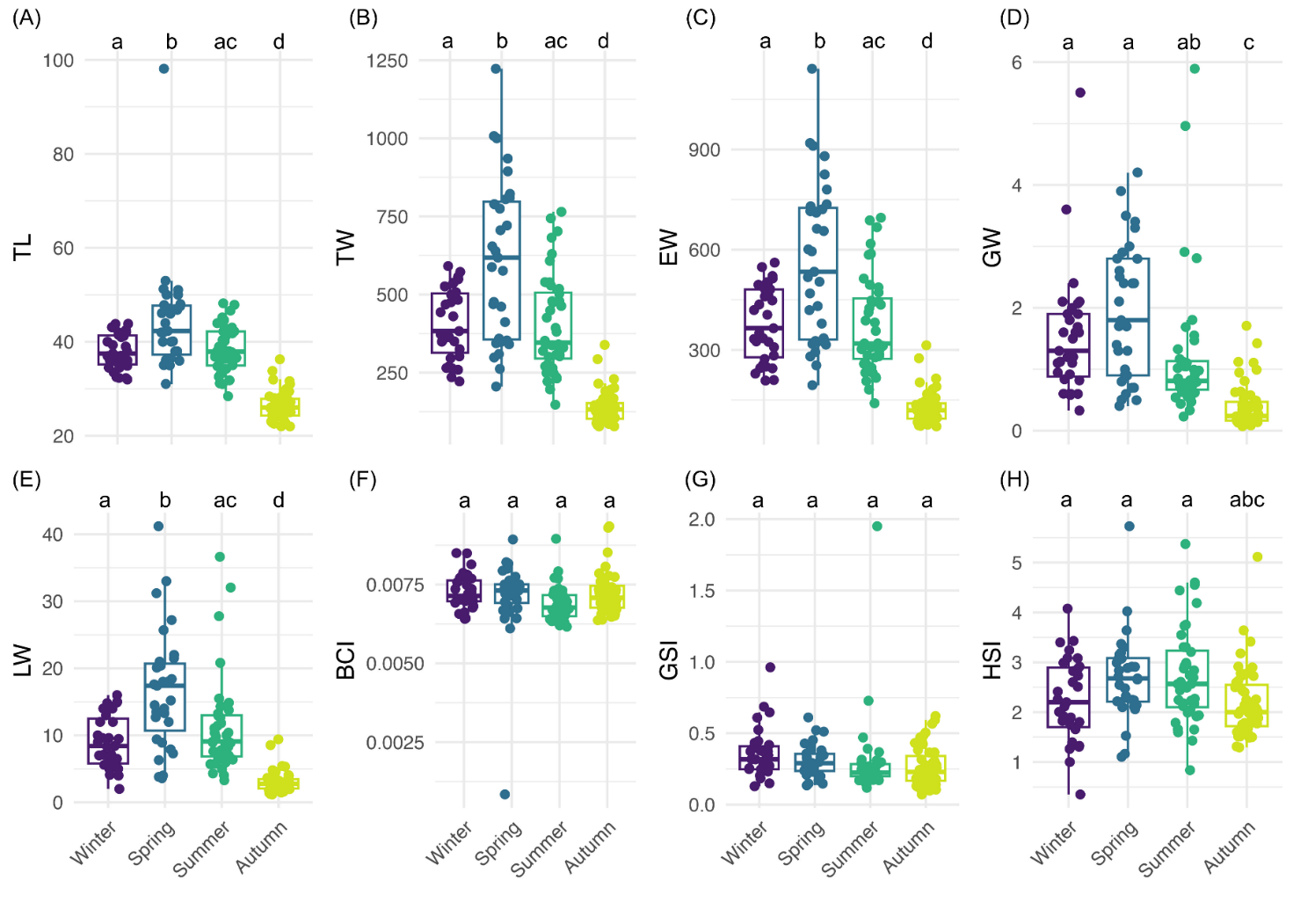


Figure S1


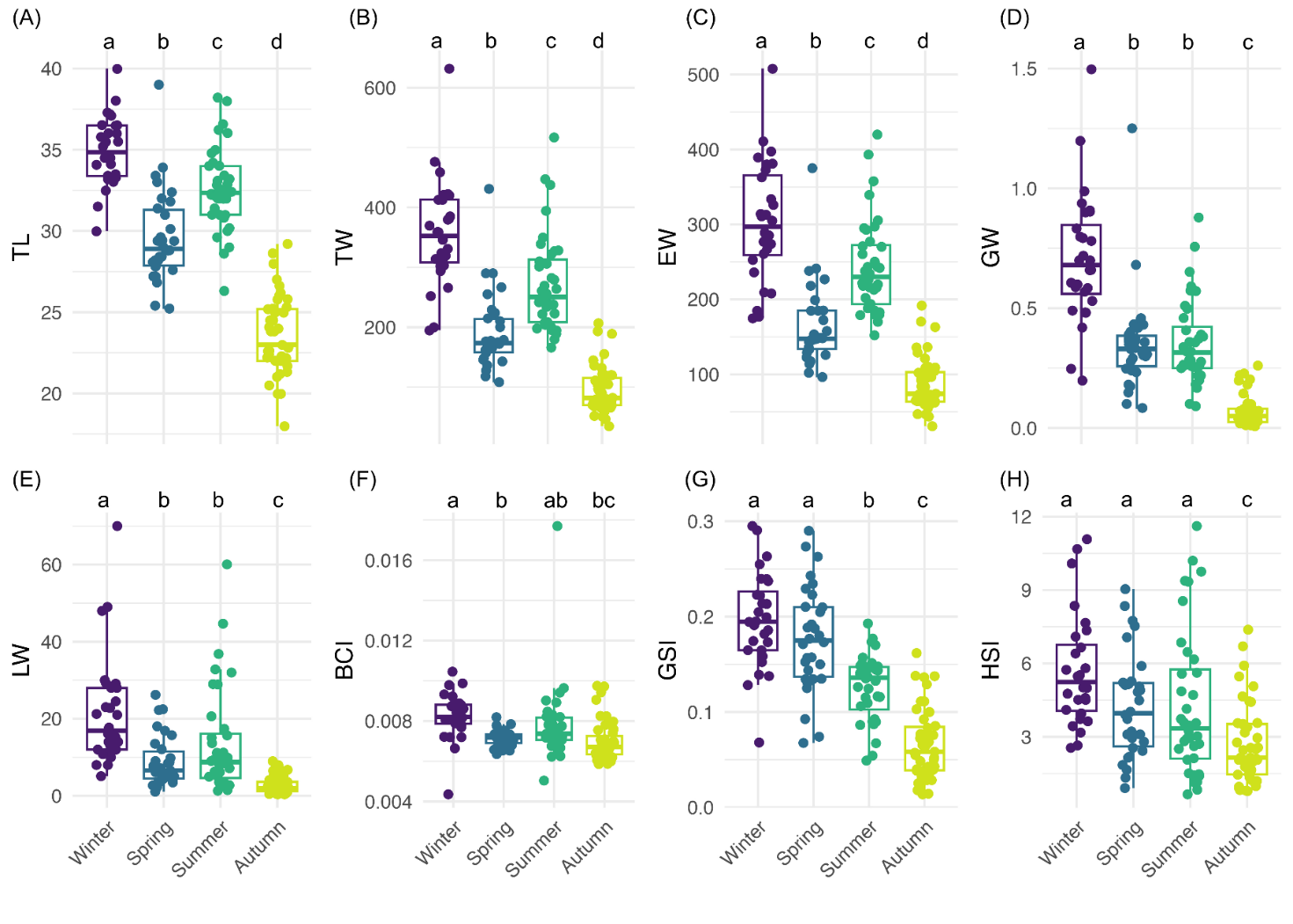


Figure S2
